# A new approach to estimating the prevalence of hereditary hearing loss: An analysis of the distribution of sign language users based on census data in Russia

**DOI:** 10.1371/journal.pone.0242219

**Published:** 2020-11-30

**Authors:** Georgii P. Romanov, Vera G. Pshennikova, Sergey A. Lashin, Aisen V. Solovyev, Fedor M. Teryutin, Aleksandra M. Cherdonova, Tuyara V. Borisova, Nikolay N. Sazonov, Elza K. Khusnutdinova, Olga L. Posukh, Sardana A. Fedorova, Nikolay A. Barashkov

**Affiliations:** 1 Laboratory of Molecular Biology, MK Ammosov North-Eastern Federal University, Yakutsk, Russia; 2 Laboratory of Molecular Genetics, Yakut Science Centre of Complex Medical Problems, Yakutsk, Russia; 3 Federal Research Center Institute of Cytology and Genetics, Siberian Branch of the Russian Academy of Sciences, Novosibirsk, Russia; 4 Novosibirsk State University, Novosibirsk, Russia; 5 Laboratory of the Human in the Arctic, Institute for Humanitarian Research and North Indigenous Peoples Problems, Federal Research Centre “The Yakut Scientific Centre of the Siberian Branch of the Russian Academy of Sciences”, Yakutsk, Russia; 6 Laboratory of Human Molecular Genetics, Institute of Biochemistry and Genetics, Ufa Federal Research Centre of the Russian Academy of Sciences, Ufa, Russia; 7 Department of Genetics and Fundamental Medicine, Bashkir State University, Ufa, Russia; University of Iowa, UNITED STATES

## Abstract

The absence of comparable epidemiological data challenges the correct estimation of the prevalence of congenital hearing loss (HL) around the world. Sign language (SL) is known as the main type of communication of deaf people. We suggest that the distribution of SL can be interpreted as an indirect indicator of the prevalence of congenital HL. Since a significant part of congenital HL is due to genetic causes, an assessment of the distribution of SL users can reveal regions with an extensive accumulation of hereditary HL. For the first time, we analyzed the data on the distribution of SL users that became available for the total population of Russia by the 2010 census. Seventy-three out of 85 federal regions of Russia were ranked into three groups by the 25^th^ and 75^th^ percentiles of the proportion of SL users: 14 regions—“low proportion”; 48 regions—“average proportion”; and 11 regions—“high proportion”. We consider that the observed uneven prevalence of SL users can reflect underlying hereditary forms of congenital HL accumulated in certain populations by specific genetic background and population structure. At least, the data from this study indicate that the highest proportions of SL users detected in some Siberian regions are consistent with the reported accumulation of specific hereditary HL forms in indigenous Yakut, Tuvinian and Altaian populations.

## Introduction

Hearing loss (HL) is one of the most common sensory disorders that makes it a serious public health problem. According to the World Health Organization (WHO), the number of people with disabling HL is approximately 466 million worldwide, including 34 million children [1]. The WHO data are based on the Global Burden of Diseases (GBD) project, which has estimated the HL prevalence based on only 42 studies in 29 countries [2]. Among many studies on HL prevalence in the general population, only a small number of surveys are suitable for analysis, and more population-based surveys are needed in all regions of the world [2, 3]. For countries with missing or scarce data, the GBD calculates the HL prevalence using a Bayesian hierarchical model that is effective for sparse data [2]. Thus, uniform description of HL prevalence is now available for only particular populations or regions since different studies are based on highly specific data and resources.

Universal newborn hearing screening (UNHS) programs are potentially the best source of data on the prevalence of congenital deafness [4–6]. The rate of congenitally deaf infants is generally considered to vary from 1 to 3 per 1,000 newborns in developed countries and up to 24 per 1,000 newborns in developing countries due to the higher presence of risk factors [4–9]. Despite all benefits of the UNHS data, a very limited number of countries (mostly highly developed countries) have massively adopted such programs, and the reported rates of congenital deafness are not applicable for other parts of the world [4, 7, 9]. The lack of systematic reporting and analysis of data also greatly affects the correct evaluation of congenital HL prevalence by the UNHS [4, 7, 9].

At the same time, there are studies assessing HL prevalence by other approaches in particular countries. One of the most well known is the 1972 National Census of the Deaf Population (NCDP) in the USA, which gathered information on the size, geographic distribution, and characteristics of the deaf population in the USA [10]. However, it is difficult to assess whether the proportion of deaf people has been the same since 1972 because the NCDP was conducted only once. Currently, approximately 5–6% of the whole population of the USA is estimated to be deaf or hard of hearing [11, 12]. These data come from the national household surveys conducted by the federal government: the Behavioral Risk Factor Surveillance System (BRFSS), the National Health Interview Survey (NHIS), and the Survey of Income and Program Participation (SIPP). Despite certain general limitations in methodology, such as difficulties in sampling a rare population, the use of a telephone-mediated survey (BRFSS), the exclusion of a certain age or social group (SIPP), and unclear distinction between deaf and hard-of-hearing individuals (NHIS), these surveys are currently the only basis of estimation of HL prevalence in the USA [11, 12].

In Italy, the data from the Italian National Institute of Social Insurance (INPS) were applied as a basis for estimating the prevalence of HL [13]. As, in Italy, sickness benefits are granted to recognized cases, the INPS keeps a record of all cases of prelingual deafness (60 dB or more for 0.5-, 1.0- and 2.0-kHz frequency tones in the better ear). The analysis of available data from 18 of 20 regions of Italy (98.2% of Italian population) detected a total of 40,887 cases of prelingual profound sensorineural HL in Italy in 2003 (the prevalence rate of 0.72 per 1,000) with differences by sex and high accumulation in the southern regions, possibly due to the epidemic incidence of maternal rubella in the 40s and the 50s and due to frequent consanguineous marriages in the past [13]. Unfortunately, there are no other studies suitable for comparison with these data.

In this work, we propose to consider the data on the prevalence of the knowledge of sign language (SL) as an alternative indicator of the prevalence of congenital and prelingual HL. Congenital or early-onset HL is the most socially significant form of HL, greatly affecting the healthy development and socialization of deaf people, most of whom use SL as their only way of communication. Therefore, a high incidence of SL users in certain locations might indicate the accumulation of hereditary forms of deafness, as half of the congenital HL cases have a genetic etiology [5]. For the first time, the data on SL users have become available as the result of the 2010 total population census in Russia.

The prevalence of congenital HL in Russia was described previously only for certain populations either by broad surveys on inherited diseases [14–16] or by molecular genetic studies performed on specific samples of deaf patients [17–33]. In this study, we present the estimation of the prevalence of hereditary severe or profound HL in Russia based on the analysis of the 2010 census data concerning the knowledge of sign language.

## Materials and methods

To analyze the prevalence of SL users in Russia, we used the data of the last (2010) comprehensive nationwide population census conducted simultaneously according to a unified statistical methodology in all regions of Russia and published by the Federal State Statistics Service (FSSS) of the Russian Federation [34]. The reports were published in thematic volumes: “Size and distribution of the population”, “Age, sex and marital status”, “Education”, “Ethnic composition and language skills, citizenship”, “Sources of livelihood”, “Number and composition of households”, “Economically active and economically inactive population”, “Length of residence of the population in the place of permanent residence”, “Housing conditions of the population”, “Fertility”, which were published as accordingly named volumes of report. The published results of the census are available separately for each region of Russia by local offices of FSSS which were aggregated in total comprehensive statistical report.

In 2010, the Russian Federation constitutionally consisted of 85 federal subjects, and the total population amounted to 142,946,788 people [34]. A distinctive feature of the 2010 census questionnaires that made this study possible was the inclusion of SL on the issue of the knowledge and use of languages. Data on the number of people reporting SL knowledge were extracted from the report tables available on the FSSS website [34]. The data in these tables were formed on the basis of the answers to the questions in the census form: “9.1 Do you speak Russian?”, where the respondents choose “yes” or “no” by putting a mark in the corresponding checkbox, and “9.2 What other languages do you speak?”, where respondents could indicate up to three languages other than Russian, and indicate knowledge of SL by putting a mark in a “sign language” checkbox. Data provided by regional departments of the FSSS were available in full for 73 out of 85 federal subjects of Russia and were analyzed to study the regional distribution of SL users. For each value, we calculated the 95% confidence interval (CI) using the BETAINV function in the Microsoft Excel table made by Mait Metspalu (Estonian Biocentre, Estonia). To categorize the data, we calculated the median, 25^th^ and 75^th^ percentiles for available values (the number and proportion of SL users). We divided regional values for SL users into three groups by lower and upper quartiles. Values significantly lower than the 25^th^ percentile were considered “low”, values significantly higher than the 25^th^ percentile and lower than the 75^th^ percentile were considered “average”, and values significantly higher than the 75^th^ percentile were considered “high”.

This study was approved by the local Biomedical Ethics Committee of Federal State Budgetary Scientific Institution “Yakut Science Centre of Complex Medical Problems”, Yakutsk, Russia (Protocol No. 16, April 16, 2015). The data were analyzed anonymously, and no informed consent forms were required.

## Limitations

It should be noted that the number of SL users cannot be directly interpreted as the number of hard-of-hearing people since a certain part of hearing people also know and use SL (relatives, social workers, interpreters, teachers). Nevertheless, we suggest that the proportion of hearing SL users can be neglected when analyzing the prevalence of SL across regions of Russia due to a relatively uniform distribution of educational institutes and accessibility of social services for deaf people in federal subjects of Russia.

## Results

**Fig 1:**
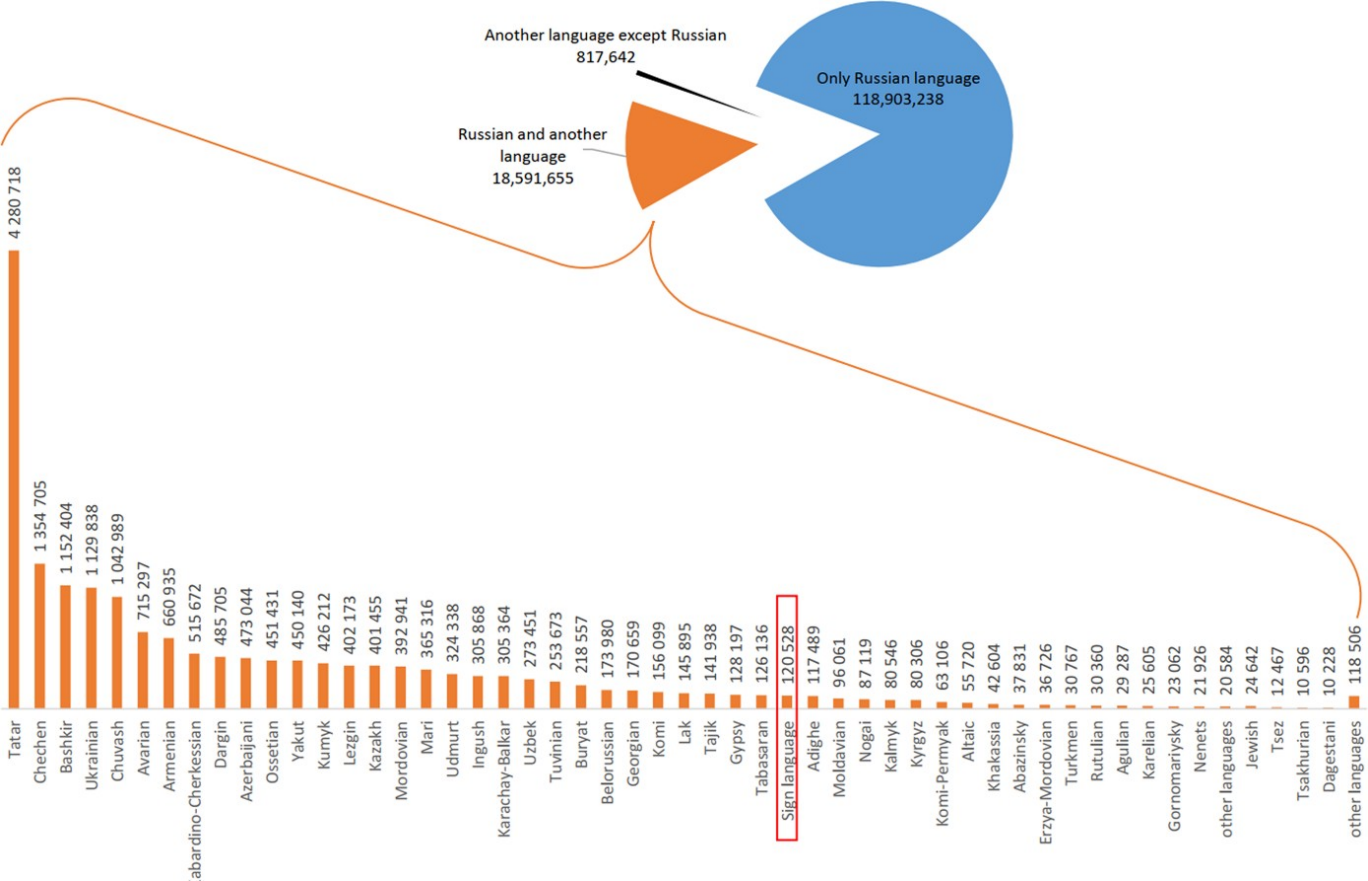
Distribution of different languages used in Russia according to the number of respondents (the 2010 census).

According to the final report of the 2010 census, 138,312,535 people answered the question about their language knowledge. Among them, 18,591,655 respondents indicated that they know and use another language besides Russian (Fig 1). In total, there are 172 languages spoken in Russia, although some of them are dialects, but, as having been specified, still counted as a language [34]. Fifty-three of them are considered foreign, such as English, French, German, Spanish, Japanese, Chinese, and other foreign languages. By excluding these languages, we counted 119 languages that respondents indicated as their native language, and SL, according to the number of SL users, ranks thirty-second in this list (Fig 1 and S1 Table).

To study the prevalence of SL users, we had two sets of relevant data: aggregated statistics and regional reports of local offices of the FSSS. According to the aggregated statistics from the census results, there were 120,528 SL users among 138,312,535 respondents, with a proportion of 0.087%. Information on SL users reported by local FSSS offices was available for 73 out of 85 federal subjects of Russia, and in this case, the total number of SL users was 107,064 out of 122,527,891 respondents, which corresponds to the same proportion of 0.087%. We assume that the “missing” 13,464 SL users are uniformly distributed among 12 regions with unavailable census data.

We analyzed the regional distribution of the number and proportion of SL users across different regions of Russia. According to the number of SL users, the regions of Russia were divided (by lower and upper quartiles) into three groups: “low number” - 19 regions; “average number” - 36 regions; and “high number” (18 regions). There were some regions with the largest number of SL users: Moscow– 9,342; Moscow Oblast’– 4,162; the Republic of Bashkortostan– 4,059; Sverdlovsk Oblast’– 3,887; Chelyabinsk Oblast’– 3,732; Rostov Oblast’– 3,557; and Saint-Petersburg– 3,553 (S1 Fig and S2 Table). The regions with the lowest number of SL users were Chukotka Autonomous Okrug– 29; Evrei Autonomous Oblast’– 111; Magadan Oblast’– 161; Kamchatka Krai– 215; Sevastopol– 243; Yamal-Nenets Autonomous Okrug– 255; and the Republic of Altai– 284 (S1 Fig and S2 Table).

**Fig 2:**
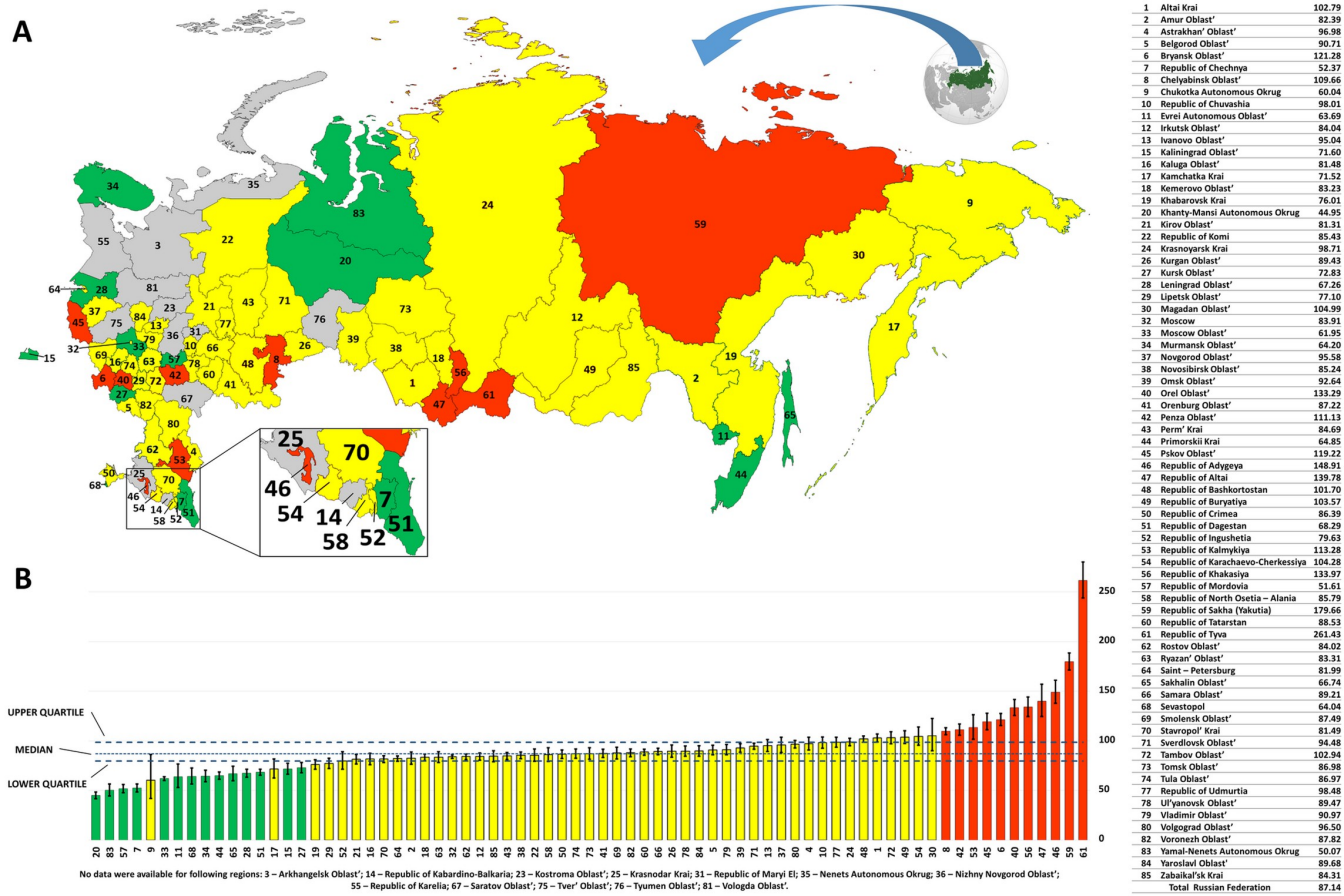
The rates of SL users in different regions of Russia. (A) Proportion of SL users across federal regions of Russia. The regions without available information are shown in gray. (B) Comparison of the proportion of SL users in different regions of Russia. The regions are shown in three different colors according to the proportion values of SL users: high values (above the 75^th^ percentile) in red, average values (between the 25^th^ and the 75^th^ percentile) in yellow, and low values (under the 25^th^ percentile) in green.

The proportion of SL users varied across federal regions of Russia, from 0.045% to 0.261%. We subdivided all regions into three categories according to the proportion of SL users: “low proportion”– 14 regions; “average proportion”– 48 regions; and “high proportion”– 11 regions (Fig 2 and S2 Table). The highest proportion of SL users was registered in the Republic of Tyva (Fig 2A)– 0.261% (CI– 0.244–0.028%). Other regions with a high proportion of SL users were as follows: the Republic of Sakha– 0.180% (CI– 0.171–0.188%); the Republic of Adygeya– 0.149% (CI– 0.138–0.161%); the Republic of Altai– 0.140% (CI– 0.124–0.157%); the Republic of Khakasiya– 0.134% (CI– 0.124–0.144%); and Orel Oblast’– 0.133% (CI– 0.125–0.142%). The lowest proportion of SL users– 0.045% (CI– 0.042–0.049%)–was registered in Khanty-Mansi Autonomous Okrug, although there were no significant differences compared to some other regions: Yamal-Nenets Autonomous Okrug (0.050%, CI– 0.044–0.057%); the Republic of Mordovia (0.052%, CI– 0.048–0.056%); the Republic of Chechnya (0.052%, CI– 0.049–0.057%); and Chukotka Autonomous Okrug (0.060%, CI– 0.041–0.086%). Some other regions also had overlapping CIs (Fig 2 and S2 Table).

## Discussion

In this study, by using census data, we determined the proportion and regional distribution of SL users in the total population (~ 140 million people) of Russia. Sign language, according to the total number of SL users, is in the thirty-second position in the list of 119 different native languages used in Russia (Fig 1 and S1 Table). The total number of SL users in Russia in 2010 was 120,528 (0.087%) out of 138,312,535 respondents, and the proportion of SL users varied from 0.045% to 0.261% in different regions of Russia.

The number of SL users varied from 29 in Chukotka Autonomous Okrug to 9,342 in Moscow, with a mean number of 1,466.63 per region, and corresponded to the number of respondents in the studied regions (S2 Table). The number of SL users in regions with a high number of respondents was also high, as expected. For example, the proportion of SL users in Moscow (0.084%, 9,342 out of 11,133,239 respondents) is close to the general proportion of SL users in Russia (0.087%). Similarly, in other regions with over 3,000,000 respondents, the proportion of SL users varied from 0.062% to 0.102% (S2 Table). These data can be relevant for health organizers planning the work of social security authorities and state health institutions.

To identify regions with values different from the general proportion of SL users in Russia (0.087%), we divided them into three groups by lower and upper quartiles: “low proportion” (14 regions), “average proportion” (48 regions), and “high proportion” (11 regions) (Fig 2 and S2 Table). The highest proportions of SL users were revealed in the Republic of Tyva (0.261%) and the Republic of Sakha (Yakutia) (0.180%). The values for the Republic of Adygeya (0.149%), the Republic of Altai (0.140%), the Republic of Khakasiya (0.134%), Orel Oblast’ (0.133%), Bryansk Oblast’ (0.121%), Pskov Oblast’ (0.119%), the Republic of Kalmykiya (0.113%), Penza Oblast’ (0.111%), and Chelyabinsk Oblast’ (0.110%) were also above the 75^th^ percentile. Some of these regions of Russia (the Republic of Tyva, the Republic of Sakha (Yakutia), the Republic of Adygeya, the Republic of Altai, the Republic of Khakasiya, and the Republic of Kalmykiya) are inhabited, in addition to Russians, by different small indigenous peoples (Tuvinians, Yakuts, Adyge, Altaians, Khakas and Kalmyks). We suggest that the observed uneven distribution of SL users in these regions can represent the underlying hereditary forms of hearing impairment accumulated by a certain genetic background, a particular ethnic history and specific demographic factors in different ethnic groups living in Russia.

Numerous studies have shown that the frequencies of Mendelian diseases and different pathogenic variants can vary significantly among different regions of Russia [35–42]. Although Russians are the major ethnic group in Russia (111 million out of the total population of 146 million), there is a significant number of indigenous ethnic populations living for hundreds of years in their historical locations (over 200 different ethnicities and ethnic groups, according to the 2010 Census) unevenly dispersed across the vast territory of Russia [34]. The small size of these populations, isolation and specific mating traditions led to significant levels of endogamy [42–47]. These factors can explain the high rates of specific hereditary pathologies detected in different regions of Russia. For example, the founder effect and long isolation of some local populations in the Volga-Ural region of Russia determined a high prevalence of autosomal-recessive osteopetrosis [35] and autosomal-recessive hypotrichosis [37]; in the Caucasus regions of Russia, monoethnic marriages led to the highest world incidence of phenylketonuria [48]; and in Eastern Siberia, the high frequency of some specific Mendelian disorders was found to be caused by the founder effect [40].

The obtained data on high proportions of SL users in some regions of Siberia (the Republic of Tyva, the Republic of Sakha (Yakutia), and the Republic of Altai) are consistent with available data on the high prevalence of unique pathogenic variants in the genes associated with hearing impairment among indigenous populations in these regions (Tuvinians, Yakuts, and Altaians) [17, 18, 25, 29, 30, 49]. We suggest that a certain accumulation of hereditary forms of hearing loss can also be detected in other regions of Russia with a high proportion of SL users.

Therefore, while SL knowledge is not defined exclusively by hearing status, the prevalence of SL users might be used as an indirect indicator of the accumulation of congenital or early-onset hereditary deafness, which, in turn, would determine the direction for more detailed genetic and epidemiological studies.

## Supporting information

S1 Fig The prevalence (in numbers) of SL users in different regions of Russia. A–Number of SL users across federal regions of Russia. The regions without available information are shown in gray. B–Comparison of the number of SL users in different regions of Russia. The regions are shown in three different colors according to the number of SL users: high values (above the 75^th^ percentile) in red, average values (between the 25^th^ and the 75^th^ percentile) in yellow, and low values (under the 25^th^ percentile) in green.
(TIF)

S1 Table The list of 119 native languages in Russia according to 138,312,535 completed questionnaires of the 2010 national census. (DOCX)

S2 Table Distribution of SL users across the Russian Federation according to the 2010 national census. (DOCX)
