# Supplementary figures and images for: A new approach to estimating the prevalence of hereditary hearing loss: An analysis of the distribution of sign language users based on census data in Russia
Source: PLoS One. 2020 Nov 30;15(11):e0242219. doi: 10.1371/journal.pone.0242219 (PMC7703874; doi:10.1371/journal.pone.0242219)

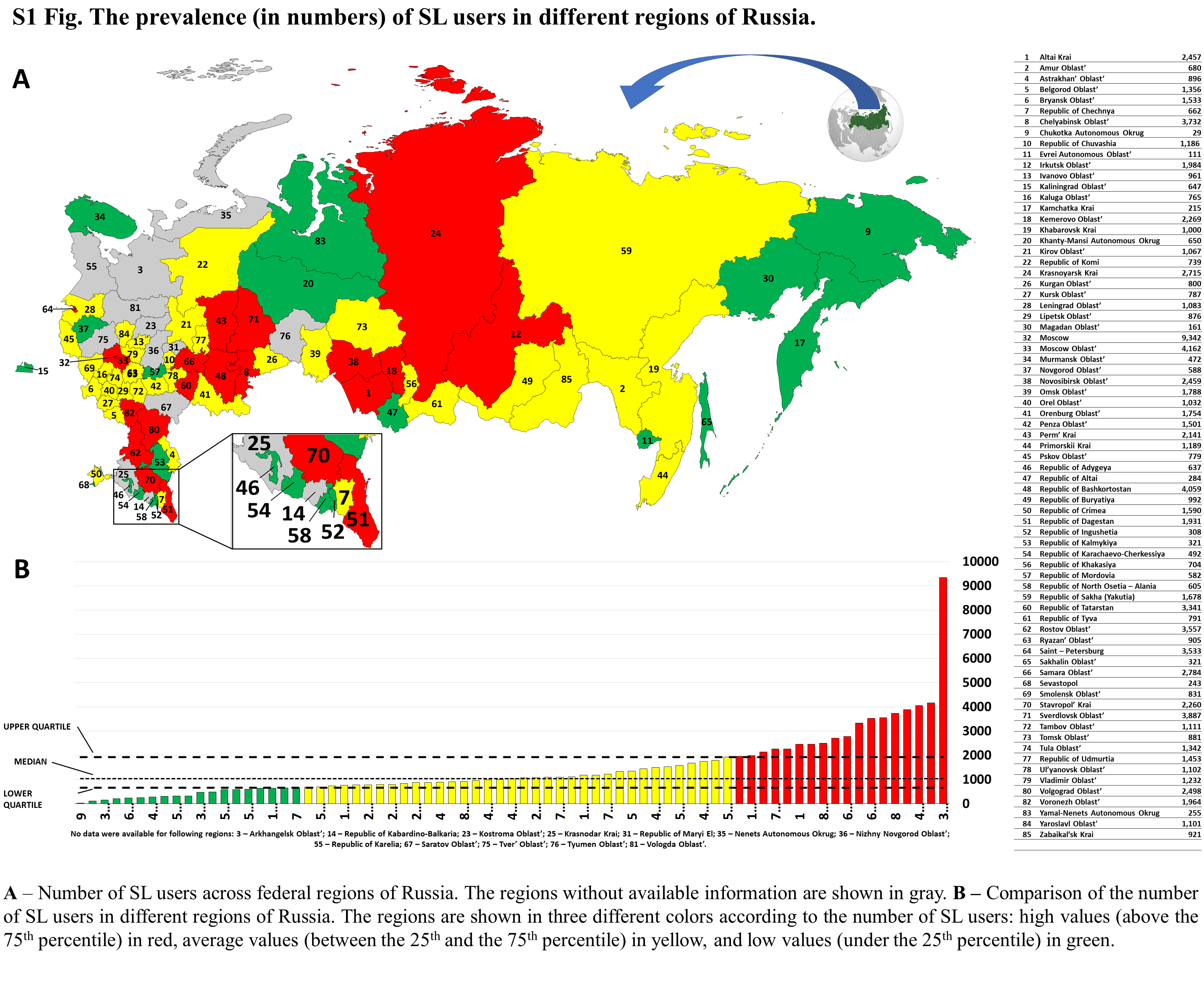

Supplement: S1 Fig — A–Number of SL users across federal regions of Russia. The regions without available information are shown in gray. B–Comparison of the number of SL users in different regions of Russia. The regions are shown in three different colors according to the number of SL users: high values (above the 75th percentile) in red, average values (between the 25th and the 75th percentile) in yellow, and low values (under the 25th percentile) in green. (TIF) [file pone.0242219.s001.tif]
