# Supplementary material for: A new approach to estimating the prevalence of hereditary hearing loss: An analysis of the distribution of sign language users based on census data in Russia
Source: PLoS One. 2020 Nov 30;15(11):e0242219. doi: 10.1371/journal.pone.0242219 (PMC7703874; doi:10.1371/journal.pone.0242219)
Supplement: S1 Table — (DOCX) [file pone.0242219.s002.docx]

**S1 Table.** **The list of 119 native languages in Russia according to 138,312,535 filled questionnaires of the 2010 national census.**

| **#** | **Language which was indicated as native** | **Number of people** | **%** |  | **#** | **Language which was indicated as native** | **Number of people** | **%** |
| --- | --- | --- | --- | --- | --- | --- | --- | --- |
| 1 | Russian | 137,494,893 | 99.41 |  | 61 | Vepsian | 3,613 | <0.01 |
| 2 | Tatar | 4,280,718 | 3.09 |  | 62 | Talysh | 3,402 | <0.01 |
| 3 | Chechen | 1,354,705 | 0.98 |  | 63 | Shor | 2,839 | <0.01 |
| 4 | Bashkir | 1,152,404 | 0.83 |  | 64 | Udi | 2,266 | <0.01 |
| 5 | Ukrainian | 1,129,838 | 0.82 |  | 65 | Tindall | 2,152 | <0.01 |
| 6 | Chuvash | 1,042,989 | 0.75 |  | 66 | Moksha-Mordovian | 2,025 | <0.01 |
| 7 | Avarian | 715,297 | 0.52 |  | 67 | Turkic | 2,025 | <0.01 |
| 8 | Armenian | 660,935 | 0.48 |  | 68 | Tat | 2,012 | <0.01 |
| 9 | Kabardino-Cherkessian | 515,672 | 0.37 |  | 69 | Adighe | 1,974 | <0.01 |
| 10 | Dargin | 485,705 | 0.35 |  | 70 | Khwarshi | 1,737 | <0.01 |
| 11 | Azerbaijani | 473,044 | 0.34 |  | 71 | Yiddish | 1,683 | <0.01 |
| 12 | Ossetian | 451,431 | 0.33 |  | 72 | Koryak | 1,665 | <0.01 |
| 13 | Yakut | 450,140 | 0.33 |  | 73 | Megrelian | 1,529 | <0.01 |
| 14 | Kumyk | 426,212 | 0.31 |  | 74 | Bagvalal | 1,447 | <0.01 |
| 15 | Lezgin | 402,173 | 0.29 |  | 75 | Nanaian | 1,347 | <0.01 |
| 16 | Kazakh | 401,455 | 0.29 |  | 76 | Alabugat-Tatar | 1,144 | <0.01 |
| 17 | Mordovian | 392,941 | 0.28 |  | 77 | Dolgan | 1,054 | <0.01 |
| 18 | Mari | 365,316 | 0.26 |  | 78 | Uigur | 1,049 | <0.01 |
| 19 | Udmurt | 324,338 | 0.23 |  | 79 | Selkup | 1,023 | <0.01 |
| 20 | Ingush | 305,868 | 0.22 |  | 80 | Hunzib | 1,012 | <0.01 |
| 21 | Karachay-Balkar | 305,364 | 0.22 |  | 81 | Esperanto | 992 | <0.01 |
| 22 | Uzbek | 273,451 | 0.20 |  | 82 | Teleut | 975 | <0.01 |
| 23 | Tuvinian | 253,673 | 0.18 |  | 83 | Archi | 970 | <0.01 |
| 24 | Buryat | 218,557 | 0.16 |  | 84 | Mansi | 938 | <0.01 |
| 25 | Belorussian | 173,980 | 0.13 |  | 85 | Karakalpak | 867 | <0.01 |
| 26 | Georgian | 170,659 | 0.12 |  | 86 | Kumandy | 738 | <0.01 |
| 27 | Komi | 156,099 | 0.11 |  | 87 | Eskimo | 508 | <0.01 |
| 28 | Lak | 145,895 | 0.11 |  | 88 | Dungan | 502 | <0.01 |
| 29 | Tajik | 141,938 | 0.10 |  | 89 | Chamalal | 500 | <0.01 |
| 30 | Gypsy | 128,197 | 0.09 |  | 90 | Tamil | 388 | <0.01 |
| 31 | Tabasaran | 126,136 | 0.09 |  | 91 | Yukaghir | 370 | <0.01 |
| **32** | **Sign language** | **120,528** | **0.09** |  | 92 | Sami | 353 | <0.01 |
| 33 | Adighe | 117,489 | 0.08 |  | 93 | Chelkan | 310 | <0.01 |
| 34 | Moldavian | 96,061 | 0.07 |  | 94 | Crimean Tatar | 308 | <0.01 |
| 35 | Nogai | 87,119 | 0.06 |  | 95 | Karata | 255 | <0.01 |
| 36 | Kalmyk | 80,546 | 0.06 |  | 96 | Tubalar | 229 | <0.01 |
| 37 | Kyrgyz | 80,306 | 0.06 |  | 97 | Ket | 213 | <0.01 |
| 38 | Komi-Permyak | 63,106 | 0.05 |  | 98 | Akhvah | 210 | <0.01 |
| 39 | Altaic | 55,720 | 0.04 |  | 99 | Botlikhian | 206 | <0.01 |
| 40 | Khakassia | 42,604 | 0.03 |  | 100 | Nivh | 198 | <0.01 |
| 41 | Abazinsky | 37,831 | 0.03 |  | 101 | Ulch | 154 | <0.01 |
| 42 | Erzya-Mordovian | 36,726 | 0.03 |  | 102 | Godoberi | 128 | <0.01 |
| 43 | Turkmen | 30,767 | 0.02 |  | 103 | Nganasan | 125 | <0.01 |
| 44 | Rutulian | 30,360 | 0.02 |  | 104 | Izhora | 123 | <0.01 |
| 45 | Agulian | 29,287 | 0.02 |  | 105 | Udege | 103 | <0.01 |
| 46 | Karelian | 25,605 | 0.02 |  | 106 | Tofalar | 93 | <0.01 |
| 47 | Gornomariysky | 23,062 | 0.02 |  | 107 | Itelmen | 82 | <0.01 |
| 48 | Nenets | 21,926 | 0.02 |  | 108 | Negidal | 74 | <0.01 |
| 49 | Jewish | 24,642 | 0.02 |  | 109 | Votic | 68 | <0.01 |
| 50 | Tsez | 12,467 | 0.01 |  | 110 | Orok | 47 | <0.01 |
| 51 | Tsakhurian | 10,596 | 0.01 |  | 111 | Aleutian | 45 | <0.01 |
| 52 | Dagestani | 10,228 | 0.01 |  | 112 | Chulym | 44 | <0.01 |
| 53 | Khanty | 9,584 | 0.01 |  | 113 | Enets | 43 | <0.01 |
| 54 | Abkhazian | 6,786 | <0.01 |  | 114 | Alyutor | 25 | <0.01 |
| 55 | Bezhtinskiy | 6,072 | <0.01 |  | 115 | Kerek | 10 | <0.01 |
| 56 | Gagauzian | 5,917 | <0.01 |  | 116 | Oroch | 8 | <0.01 |
| 57 | Andean | 5,800 | <0.01 |  | 117 | Sirenik | 5 | <0.01 |
| 58 | Even | 5,656 | <0.01 |  | 118 | Yuit | 4 | <0.01 |
| 59 | Chukchi | 5,095 | <0.01 |  | 119 | Yugh | 1 | <0.01 |
| 60 | Evenki | 4,802 | <0.01 |  |  |  |  |  |
