# Supplementary material for: A new approach to estimating the prevalence of hereditary hearing loss: An analysis of the distribution of sign language users based on census data in Russia
Source: PLoS One. 2020 Nov 30;15(11):e0242219. doi: 10.1371/journal.pone.0242219 (PMC7703874; doi:10.1371/journal.pone.0242219)
Supplement: S2 Table — (DOCX) [file pone.0242219.s003.docx]

**S2 Table.** **Distribution of SL users across the Russian Federation according to the 2010 national census.**

| # | Federal regions | Total number of respondents | Number of SL users | Proportion of SL users | 0.95 Credible Interval (%) |
| --- | --- | --- | --- | --- | --- |
| 1 | Altai Krai | 2,390,238 | 2,457 | 0.103% | (98.81 - 106.94) |
| 2 | Amur Oblast’ | 825,370 | 680 | 0.082% | (76.43 - 88.82) |
| 3 | Arkhangelsk Oblast’ | No data | | | |
| 4 | Astrakhan’ Oblast’ | 923,857 | 896 | 0.097% | (90.84 - 103.55) |
| 5 | Belgorod Oblast’ | 1,494,848 | 1,356 | 0.091% | (86.01 - 95.67) |
| 6 | Bryansk Oblast’ | 1,264,040 | 1,533 | 0.121% | (115.36 - 127.5) |
| 7 | Republic of Chechnya | 1,263,967 | 662 | 0.052% | (48.54 - 56.52) |
| 8 | Chelyabinsk Oblast’ | 3,403,190 | 3,732 | 0.110% | (106.2 - 113.24) |
| 9 | Chukotka Autonomous Okrug | 48,299 | 29 | 0.060% | (41.91 - 86.22) |
| 10 | Republic of Chuvashia | 1,210,067 | 1,186 | 0.098% | (92.59 - 103.75) |
| 11 | Evrei Autonomous Oblast’ | 174,281 | 111 | 0.064% | (52.92 - 76.69) |
| 12 | Irkutsk Oblast’ | 2,360,828 | 1,984 | 0.084% | (80.42 - 87.82) |
| 13 | Ivanovo Oblast’ | 1,011,126 | 961 | 0.095% | (89.23 - 101.24) |
| 14 | Republic of Kabardino-Balkaria | No data | | | |
| 15 | Kaliningrad Oblast’ | 903,598 | 647 | 0.072% | (66.3 - 77.34) |
| 16 | Kaluga Oblast’ | 938,883 | 765 | 0.081% | (75.91 - 87.46) |
| 17 | Kamchatka Krai | 300,611 | 215 | 0.072% | (62.59 - 81.74) |
| 18 | Kemerovo Oblast’ | 2,726,229 | 2,269 | 0.083% | (79.88 - 86.72) |
| 19 | Khabarovsk Krai | 1,315,596 | 1,000 | 0.076% | (71.45 - 80.87) |
| 20 | Khanty-Mansi Autonomous Okrug | 1,446,106 | 650 | 0.045% | (41.63 - 48.54) |
| 21 | Kirov Oblast’ | 1,312,237 | 1,067 | 0.081% | (76.58 - 86.34) |
| 22 | Republic of Komi | 865,085 | 739 | 0.085% | (79.49 - 91.81) |
| 23 | Kostroma Oblast’ | No data | | | |
| 24 | Krasnoyarsk Krai | 2,750,347 | 2,715 | 0.099% | (95.07 - 102.5) |
| 25 | Krasnodar Krai | No data | | | |
| 26 | Kurgan Oblast’ | 894,533 | 800 | 0.089% | (83.45 - 95.85) |
| 27 | Kursk Oblast’ | 1,080,604 | 787 | 0.073% | (67.92 - 78.1) |
| 28 | Leningrad Oblast’ | 1,610,061 | 1,083 | 0.067% | (63.38 - 71.39) |
| 29 | Lipetsk Oblast’ | 1,136,201 | 876 | 0.077% | (72.16 - 82.38) |
| 30 | Magadan Oblast’ | 153,343 | 161 | 0.105% | (90.01 - 122.51) |
| 31 | Republic of Maryi El | No data | | | |
| 32 | Moscow | 11,133,239 | 9,342 | 0.084% | (82.23 - 85.63) |
| 33 | Moscow Oblast’ | 6,717,809 | 4,162 | 0.062% | (60.1 - 63.87) |
| 34 | Murmansk Oblast’ | 735,158 | 472 | 0.064% | (58.67 - 70.26) |
| 35 | Nenets Autonomous Okrug | No data | | | |
| 36 | Nizhny Novgorod Oblast’ | No data | | | |
| 37 | Novgorod Oblast’ | 615,171 | 588 | 0.096% | (88.17 - 103.63) |
| 38 | Novosibirsk Oblast’ | 2,884,805 | 2,459 | 0.085% | (81.94 - 88.68) |
| 39 | Omsk Oblast’ | 1,930,086 | 1,788 | 0.093% | (88.45 - 97.03) |
| 40 | Orel Oblast’ | 774,250 | 1,032 | 0.133% | (125.41 - 141.67) |
| 41 | Orenburg Oblast’ | 2,010,970 | 1,754 | 0.087% | (83.24 - 91.4) |
| 42 | Penza Oblast’ | 1,350,714 | 1,501 | 0.111% | (105.65 - 116.89) |
| 43 | Perm’ Krai | 2,527,983 | 2,141 | 0.085% | (81.18 - 88.36) |
| 44 | Primorskii Krai | 1,833,419 | 1,189 | 0.065% | (61.27 - 68.64) |
| 45 | Pskov Oblast’ | 653,439 | 779 | 0.119% | (111.14 - 127.88) |
| 46 | Republic of Adygeya | 427,776 | 637 | 0.149% | (137.8 - 160.93) |
| 47 | Republic of Altai | 203,180 | 284 | 0.140% | (124.46 - 157) |
| 48 | Republic of Bashkortostan | 3,991,097 | 4,059 | 0.102% | (98.62 - 104.88) |
| 49 | Republic of Buryatiya | 957,776 | 992 | 0.104% | (97.33 - 110.22) |
| 50 | Republic of Crimea | 1,840,435 | 1,590 | 0.086% | (82.25 - 90.74) |
| 51 | Republic of Dagestan | 2,827,689 | 1,931 | 0.068% | (65.31 - 71.4) |
| 52 | Republic of Ingushetia | 386,767 | 308 | 0.080% | (71.24 - 89.04) |
| 53 | Republic of Kalmykiya | 283,381 | 321 | 0.113% | (101.56 - 126.36) |
| 54 | Republic of Karachaevo-Cherkessiya | 471,785 | 492 | 0.104% | (95.48 - 113.91) |
| 55 | Republic of Karelia | No data | | | |
| 56 | Republic of Khakasiya | 525,493 | 704 | 0.134% | (124.44 - 144.23) |
| 57 | Republic of Mordovia | 1,127,644 | 582 | 0.052% | (47.59 - 55.98) |
| 58 | Republic of North Osetia – Alania | 705,213 | 605 | 0.086% | (79.23 - 92.9) |
| 59 | Republic of Sakha (Yakutia) | 933,985 | 1,678 | 0.180% | (171.28 - 188.46) |
| 60 | Republic of Tatarstan | 3,774,032 | 3,341 | 0.089% | (85.58 - 91.58) |
| 61 | Republic of Tyva | 302,567 | 791 | 0.261% | (243.87 - 280.27) |
| 62 | Rostov Oblast’ | 4,233,568 | 3,557 | 0.084% | (81.3 - 86.83) |
| 63 | Ryazan’ Oblast’ | 1,086,357 | 905 | 0.083% | (78.06 - 88.91) |
| 64 | Saint – Petersburg | 4,308,803 | 3,533 | 0.082% | (79.34 - 84.74) |
| 65 | Sakhalin Oblast’ | 480,996 | 321 | 0.067% | (59.83 - 74.45) |
| 66 | Samara Oblast’ | 3,120,626 | 2,784 | 0.089% | (85.96 - 92.59) |
| 67 | Saratov Oblast’ | No data | | | |
| 68 | Sevastopol | 379,442 | 243 | 0.064% | (56.49 - 72.62) |
| 69 | Smolensk Oblast’ | 949,813 | 831 | 0.087% | (81.75 - 93.64) |
| 70 | Stavropol’ Krai | 2,773,452 | 2,260 | 0.081% | (78.2 - 84.92) |
| 71 | Sverdlovsk Oblast’ | 4,114,267 | 3,887 | 0.094% | (91.55 - 97.49) |
| 72 | Tambov Oblast’ | 1,079,290 | 1,111 | 0.103% | (97.07 - 109.17) |
| 73 | Tomsk Oblast’ | 1,012,894 | 881 | 0.087% | (81.43 - 92.91) |
| 74 | Tula Oblast’ | 1,543,017 | 1,342 | 0.087% | (82.45 - 91.75) |
| 75 | Tver Oblast’ | No data | | | |
| 76 | Tyumen Oblast’ | No data | | | |
| 77 | Republic of Udmurtia | 1,475,411 | 1,453 | 0.098% | (93.55 - 103.68) |
| 78 | Ul’yanovsk Oblast’ | 1,231,734 | 1,102 | 0.089% | (84.34 - 94.91) |
| 79 | Vladimir Oblast’ | 1,354,266 | 1,232 | 0.091% | (86.04 - 96.19) |
| 80 | Volgograd Oblast’ | 2,588,723 | 2,498 | 0.096% | (92.79 - 100.35) |
| 81 | Vologda Oblast’ | No data | | | |
| 82 | Voronezh Oblast’ | 2,236,446 | 1,964 | 0.088% | (84.02 - 91.79) |
| 83 | Yamal-Nenets Autonomous Okrug | 509,279 | 255 | 0.050% | (44.3 - 56.61) |
| 84 | Yaroslavl Oblast’ | 1,227,650 | 1,101 | 0.090% | (84.54 - 95.14) |
| 85 | Zabaikal’sk Krai | 1,092,449 | 921 | 0.084% | (79.04 - 89.93) |
| Total | | 138,312,535 | 120,528 | 0.087% | (86.65 - 87.63) |
